# Improving access to low vision services

**Published:** 2012

**Authors:** Peggy Pei-Chia Chiang, Jill E Keeffe

**Affiliations:** Postdoctoral research fellow, Singapore Eye Research Institute; Centre for Eye Research Australia (CERA). Email: peggychiang81@gmail.com; Director, World Health Organization Collaborating Centre for Prevention of Blindness at CERA.

**Figure:**
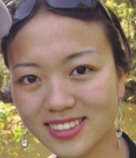
Peggy Pei-Chia Chiang

**Figure:**
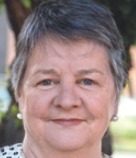
Jill E Keeffe

Our recent survey^1^, ^2^ found that low vision services were often inaccessible to large numbers of people in low- and middle-income countries.

Based on the findings of this research, we suggest three areas for action: human resources, sustainability of services, and advocacy. However, it is important to keep in mind that these strategies must be adapted to suit your situation.

## Human resources

- Integrate low vision into existing ophthalmic and optometric curricula and include it in the practical training of education and rehabilitation workers
- Offer informal low vision workshops and courses for eye care workers who have not received formal training.
- Delegate tasks to less specialised health workers where possible. For instance, instead of the optometrist doing the simple refraction and basic low vision care, a trained vision technician could do these tasks.
- Build on the skills of existing staff. For example, in areas where there are no ophthalmologists or optometrists, refractionists, ophthalmic nurses, and opticians can be trained to take on additional low vision tasks appropriate to their skills and experience.

## Sustainability

Strengthen community-based rehabilitation and outreach services.

- During outreach, you could explain or show how the home environment can be adapted and make timely referrals to district level care. Through outreach, people can be followed up to ensure they are still able to use their low vision devices, and you can give refresher lessons to those who need it. In addition, children with poor vision can be detected and supported early.
- Outreach services should be carried out on a regular basis, although the frequency may vary, depending on need.
- Integrate low vision services into existing education, rehabilitation, and eye care systems. Establish appropriate and healthy collaborations between the government and the private sector.
- Non-governmental organisations must work together with the private sector and government to support and fund low vision services. However, for this to work in the long term, the government must take the lead and take ownership of programmes and services.

## Advocacy

We recommend two strategies:

- Use strong research evidence on which to formulate policy.
- Encourage NGOs and all stakeholders with an interest in low vision to come together under one umbrella organisation, i.e. a national VISION 2020 or prevention of blindness committee. The group can then deliver the policy message with one clear voice.

Once advocacy and lobbying have started, more detailed planning must be done at the implementation level. For instance, encourage local government and policy makers to include low vision in their district VISION 2020 or eye care plans.
